# Effect of resistance training on bioelectrical phase angle in older adults: a systematic review with Meta-analysis of randomized controlled trials

**DOI:** 10.1007/s11154-022-09747-4

**Published:** 2022-08-03

**Authors:** Francesco Campa, Lucas Antonio Colognesi, Tatiana Moro, Antonio Paoli, Andrea Casolo, Leandro Santos, Rafael Ribeiro Correia, Ítalo Ribeiro Lemes, Vinícius Flávio Milanez, Diego Destro Christofaro, Edilson Serpeloni Cyrino, Luís Alberto Gobbo

**Affiliations:** 1 Department of Biomedical Sciences, University of Padua, 35131 Padova, Italy; 2 Skeletal Muscle Assessment Laboratory (LABSIM), Department of Physical Education, School of Technology and Sciences, São Paulo State University (UNESP), Presidente Prudente, 19060-900 Padova, SP Brazil; 3 Graduate Program in Physical Therapy, School of Technology and Science, São Paulo State University (UNESP), Brazil, UNESP, 19060-900 Presidente Prudente, SP Brazil; 4 Metabolism, Nutrition, and Exercise Laboratory, Physical Education and Sport Center, Londrina State University, 86057-970 Londrina, PR Brazil; 5 Multicentric Program of Postgraduate in Physiological Sciences, São Paulo State University (UNESP), School of Dentistry of Araçatuba, São Paulo, Brazil., UNESP, 19060-900 Sao Paulo, SP Brazil; 6 Department of Physical Education, Oeste Paulista University, UNOESTE, 19067-175 Presidente Prudente, SP Brazil

**Keywords:** Aging, Body composition, BIA, BIVA, Phase angle, Strength

## Abstract

Resistance training has been proposed as a valid practice to counteract the aging effect on body mass and its components, which can be easily evaluated though the bioelectrical impedance analysis. This study aimed to achieve a systematic review with meta-analysis on the impact of resistance training on bioelectrical proprieties in older adults.

A literature review was done in four electronic databases up to 1 January 2022. The inclusion criteria were: (i) participants aged ≥ 60 years; (ii) resistance training lasted ≥ 8 weeks; (iii) measurement of raw bioelectrical parameters in randomized controlled study designs.

The outcomes of the trial had to be bioelectrical phase angle (PhA), resistance (R), and reactance (Xc). The methodological quality was assessed using the Rosendal scale.

Overall, seven studies with a total of 344 participants were eligible for the analysis. The quality assessment yielded a score of 71.3%. Bioelectrical PhA (0.52 degree [95%CI 0.32, 0.71], p < 0.001) and Xc (3.58 ohms [95%CI 1.97, 5.19], p < 0.001) increased, whereas R decreased (-28.50 ohms [95%CI -41.39, -15.60], p < 0.001) after the resistance training programs.

In this meta-analysis, resistance training promoted increases of PhA, which result from an increase in Xc concomitant with a reduction in R. According to the bioimpedance vector analysis, resistance-trained people experienced a beneficial leftward vector displacement, whilst inactivity induced a rightward vector displacement within the R-Xc graph. In future, more sophisticated and rigorous studies that address specific criteria, methods and targeted designs are required to identify which equipment and protocols allow for an optimization of the resistance training effects.

**Registration code in PROSPERO**: CRD42020168057.

## Introduction

Resistance training is a form of periodic exercise whereby external weights provide a progressive overload to skeletal muscles in order to promote strength and/or muscle mass increase and other health-related adaptations [1, 2]. Resistance training has stood out as one of the more effective strategies for counteracting the remodeling in body composition caused by the aging process [2, 3]. Different training equipments (e.g., weight machines, suspension tools, and elastic tubes) progression of training loads, and periodization strategies have been proposed with the aim to optimize resistance training efficiency and suitability for older people [2, 4, 5].

Bioelectrical impedance analysis (BIA) was identified as a non-invasive, low cost, and user-friendly method for assessing body composition adaptations in response to aging and training [6, 7]. BIA is based on the measure of the bioimpedance, resulting from the conductivity proprieties ​​of the human body and the response to the passage of a sinusoidal current through its different biological components [8–10]. Current opposition is measured by the resistance (R), while current delay, due to the concept of cell membrane capacitance, results in the reactance (Xc) [8, 10]. The relationship between the two bioimpedance components, R and Xc, is expressed by the bioelectrical phase angle (PhA), a biomarker able to reflect the intra and extracellular fluid distribution, cell integrity, and nutritional status in elderly subjects [6, 11–13]. Due to its portability and practicality, BIA represents one of the main alternatives to more accurate methods for assessing body composition [14]. For this purpose, the raw bioelectrical R, Xc, and PhA are used in mathematical models and population-specific predictive equations to quantify a wide range of components [7, 14, 15]. Alternatively, body composition can be assessed through a BIA-based qualitative analysis which consists of the interpretation of the bioelectrical vector plotted in the R-Xc graph [16, 17]. Particularly, the distance from the vector to the X axis is graphically represented by the PhA and leftward or rightward displacements result in PhA increase or decrease, respectively [10]. Given that the bioelectrical impedance proprieties are mainly related to body fluid content and its distribution among compartments [18, 19], the bioimpedance vector analysis (BIVA) is informative of body composition characteristics and its adaptations due to aging and/or after a training period [7, 14] In fact, the position of the bioimpedance vector on the RXc graph reveals the PhA and provides information on cell mass and fluid status [7].

Throughout the lifespan, PhA increases progressively with age, reaching maximum values between 16 and 38 years (PhA = 7.3°, 95% CI 7.0°, 7.5°) and decreases with aging, with estimated values close to 5.4° (95% CI 5.3°, 5.6°) for elderly people over 80 years old and with higher mean values for men than women at all stages of life [20]. Recent studies indicated that PhA can be influenced by the level of physical activity and is higher in active subjects compared to sedentary people [21, 22]. In older adults, increases in PhA have been associated with a gain in intracellular water and then in muscle mass, while decreases in PhA have been found after extracellular water expansions, inflammation, and reduction in muscle mass [12, 23, 24]. The evaluation of the raw bioimpedance parameters has gained more attention than the quantification of body composition parameters in interventional studies involving older people since this practice helps avoid the typical concerns associated with the use of regression equations and assumptions of constant hydration of the fat-free mass and assumptions of constant hydration of the fat-free body. [11, 25, 26]. A common aim of the studies involving older adults into resistance training programs is to counteract the aging effect on bioelectrical proprieties, inducing increases in PhA which result in body composition and physical performance improvements [27–29].

Despite the increase of the number of studies suggesting that BIA-derived PhA has clinical relevance and it is related to health status and physical functions, there is still no consensus regarding how bioelectrical proprieties change after a resistance training program with respect to an inactivity period in older adults. In fact, there are no articles with comprehensive descriptions available which summarize the adaptations in the bioimpedance components in response to resistance training in older people. Therefore, to further clarify the influence of resistance training on R, Xc, and PhA in older people, we conducted a meta-analysis to quantify the magnitude of the outcomes relative to beneficial changes in physical function.

## Methods

This study followed the recommendations of the Preferred Reporting Items for Systematic Reviews and Meta-Analyses (PRISMA) guideline [30]. The review protocol was prospectively registered on PROSPERO with the following number: CRD42020168057.

### Eligibility criteria

For eligibility criteria, the PICOS strategy [31] was adopted: in which “P” (patients), corresponded to participants aged ≥ 60 years, of all genders and ethnicities; “I” (intervention), was designated as resistance training lasted ≥ 8 weeks, “C” (comparison), was defined as the execution of no physical exercise, “O” (outcomes), were the raw BIA-derived parameters, and “S” (study design), were related only to randomized clinical trials.

Exclusion criteria were as follows: (i) articles did not include a full-text description of the study; (ii) not in English language; (iii) not a randomized controlled trial; (iv) the intervention group received resistance training combined with other training strategies (e.g., aerobic or balance training) or nutritional supplementation, as well as those with sick individuals and/or specific health disease or recent cardiovascular events; (v) raw BIA measures not reported; and (vi) case studies, review studies, experimental models, and reply letters.

### Search strategy

Potential studies were identified by using a systematic search process and were being conducted in the following databases: PubMed, Scopus, WoS, and google scholar. The searches were carried out from inception until January 1, 2022. The search query was applied to the source title, abstract, and keywords, and included combinations of at least one of the terms identifying resistance training, with at least one of the terms identifying the bioimpedance parameters, and a term on the population of interest. The resulting search query was: (“resistance training” OR “resistance-trained” OR “RT”) AND (“BIA” OR “bioimpedance” OR “bioelectrical impedance” OR “bioelectrical impedance analysis” OR “phase angle” OR “PhA”) AND (“older adults” or “elderly people”). To identify additional relevant papers, hand searching of the reference lists of the included papers was performed.

### Study selection

Four independent reviewers performed the selection process (LAG, RRC, IRL, and VFM). An automatic filter of EndNote™ v. 20.1 (Clarivate™) was used to remove duplicates after conducting the searches. The reference lists of all included studies were hand-searched for missing publications. The same reviewers screened the remaining articles for eligibility by abstract and full text. Two investigators (DDC and ESC) reviewed any differences of opinion with the intention of making the final decision.

### Data extraction

Two reviewers (LAC and LS) performed data extraction using a specially designed Microsoft Excel spreadsheet. A third investigator (FC) participated in case of disagreements between reviewers. Data items were: first author, year of publication, country of origin, study population characteristics (e.g., sample size, gender, and age), type of intervention (e.g., duration, weekly frequency, and training protocol), BIA-device and procedures (e.g., device, technology, and sample frequency), percentage of changes in bioelectrical parameters (PhA, R, and Xc) after intervention, as well as the pre- and post-intervention values. In the event that data were not adequately reported, the corresponding author was contacted via email in an attempt to retrieve the missing data.

### Study risk of Bias Assessment

Included studies were examined for methodological quality using the Rosendal Scale [32] by two independent reviewers (LAC and LAG). The total score was determined by dividing the number of ‘yes’ responses by the total number of applicable items. Studies with an excellent methodological quality are indicated by a score ≥ 60%, while studies with a score < 50% are typically excluded from reviews owing to their increased risk of experimental bias [32]. Disagreements in the implementation of the score were solved by a third co-author (FC).

### Statistical analysis

The Review Manager program, version 5.3 (RevMan, Copenhagen: The Nordic Cochrane Center, The Cochrane Collaboration, 2014) was used for grouping, analyzing data and performing the meta-analysis. Studies that contained more than one intervention group had their populations grouped so that they could be compared with the control group. When the tests were considered sufficiently homogeneous, with an I² value less than 50%, a fixed-effect model was used, whereas when the tests were characterized as heterogeneous, with an I² value greater than 50%, it was applied a randomized effect model. The mean difference with 95% of the confidence interval (CI) was calculated.

## Results

### Study selection

**Fig. 1:**
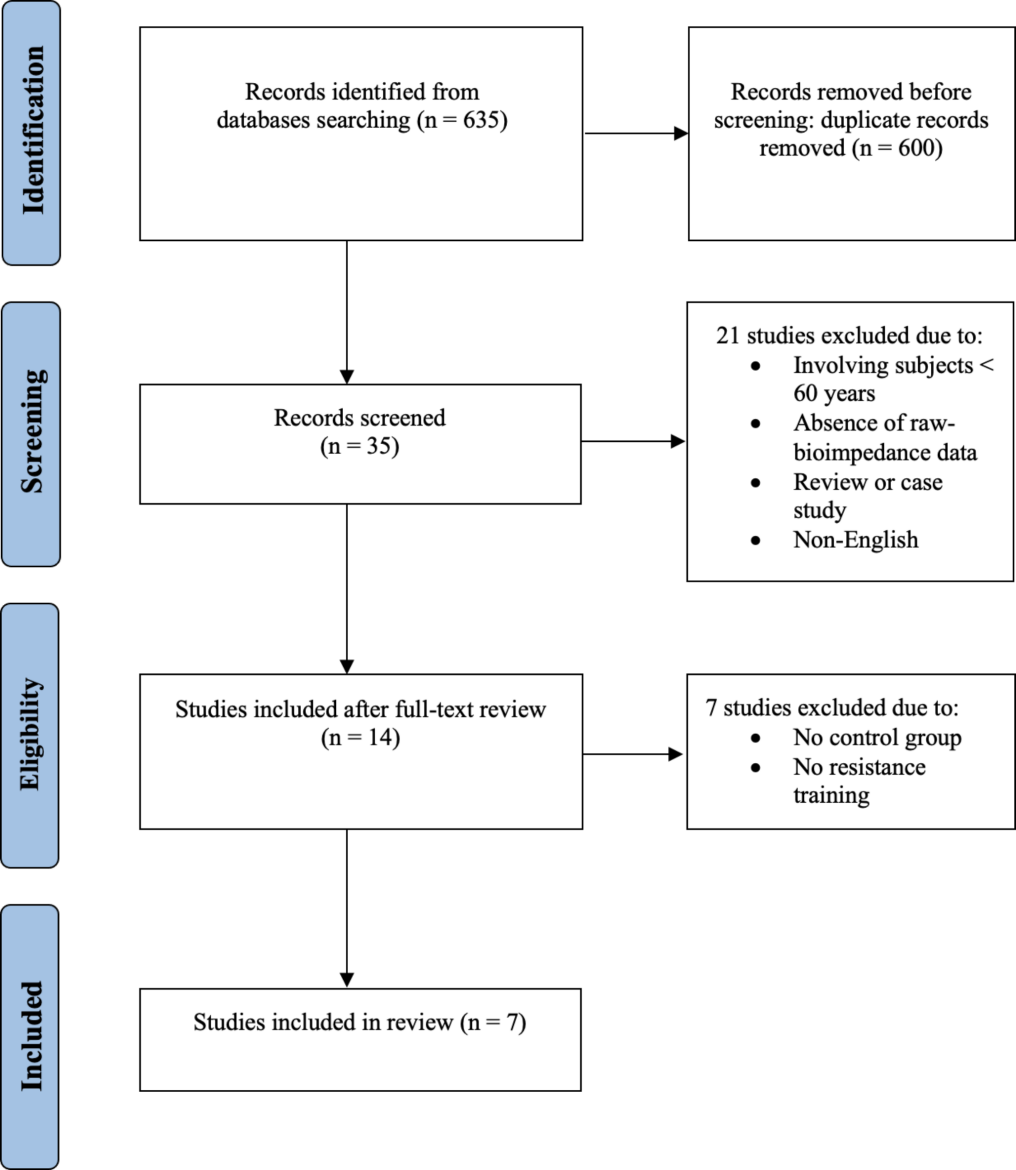
Flowchart of the included studies

The search strategy resulted in a total of 635 studies, as shown in Fig. 1. The examination of titles and abstracts, based on the inclusion and exclusion criteria, identified 14 potential eligible articles, of which seven were included in the meta-analysis after reading the full texts [28, 33–38]. Lack of resistance training or control group excluded seven studies [5, 27, 29, 39–42].

### Quality assessment

Methodological quality assessment yielded an average Rosendal score of 71.3 ± 6.6%; all trials scored ≥ 50% (range 64–83%). Results of the quality assessment are shown in Supplementary Table S1.

### Characteristics and results of the included studies

A total of 344 healthy individuals of both sexes were included in the meta-analysis. Most studies were conducted in Brazil (n = 5) [34–38], with only two studies conducted in another country (Italy) [28, 33]. Four studies had more than one intervention group [33, 34, 36, 38], with their data then grouped for comparison with a control group. The training period varied between 8 and 12 weeks, and all studies performed a progressive increase in load or intensity, as the study progressed. The other characteristics of each article included in the meta-analysis are shown in Table 1. The values ​​of PhA, R, and Xc before and after intervention, as well as their magnitude of change (effect size = ES), are described in Table 2.

**Table 1:** General characteristics of the studies selected for the meta-analysis

First Author, Year	Country	Gender	Sample size	Age, yrs (Mean ± SD)	Duration and frequency	Protocol, sets x reps	BIA-device and procedures	Significant changes after intervention
Tomeleri CM, 2018	Brazil	Female	51	70.6 ± 5.1	12 weeks, 3x	8 exercises 3 × 10-15	Hydra 4200, Xitron technologies, San Diego, USA; foot to hand technology at 50 kHz	↑ PhA = + 0.4°, + 7.4% ↓ R = N.A. ↑ Xc = N.A.
Cunha PM, 2018	Brazil	Female	62	68.6 ± 5.0	12 weeks, 3x	8 exercises G1S: 1 × 10-15 G3S: 3 × 10-15	Hydra 4200, Xitron technologies, San Diego, USA; foot to hand technology at 50 kHz	↑ PhA (G1S = + 0.3°, + 4.8%; G3S = + 0.4° +7.4%) ↓ R (G1S = -24.2 Ω, -4.9%; G3S = -32.6 Ω, 5.3%) ↑ Xc (G1S = 3.2 Ω, + 6.1%; G3S = + 3.8 Ω, + 6.8%)
Campa F, 2018	Italy	Female	30	66.1 ± 4.7	12 weeks, 2x	6 exercises 4 × 12	101 Anniversary, AKERN Systems, Florence, Italy; foot to hand technology at 50 kHz	↑ PhA = + 0.3°, + 7.2% ↓ R = -15 Ω, -3.2% ↑ Xc = + 3.5 Ω, + 7.1%
Ribeiro AS, 2017	Brazil	Female	76	68.5 ± 5.7	12 weeks, 3x	8 exercises GCC: 3 × 8–12 GCP: 3 × 12-10-8	Hydra 4200, Xitron technologies, San Diego, USA; foot to hand technology at 50 kHz	↑ PhA (CLG = + 0.1°, + 2.9%; PLG = 0.2°, + 4.1%) ↓ R (CLG = -21.6 Ω, -3.7%; PLC = -14.9 Ω, 2.6%) ↑ Xc (CLG = + 1.9 Ω, + 3.4%; PLG = + 4 Ω, + 7.4%)
Souza MF, 2017	Brazil	Female	41	67.2 ± 4.5	12 weeks, 3x	8 exercises 3 × 10-15	Hydra 4200, Xitron technologies, San Diego, USA; foot to hand technology at 50 kHz	↑ PhA = + 0.4°, + 6.5% ↓ R = -19.1 Ω, -3.2%
Dos Santos L, 2020	Brazil	Female	59	67.3 ± 4.4	8 weeks, 3x	8 exercises NG: 3 × 12/10/8 WG: 3 × 15/10/5	Hydra 4200, Xitron technologies, San Diego, USA; foot to hand technology at 50 kHz	↑ PhA (NG = + 0.3°, + 5.3%; WG = + 0.6°, + 10.6%) ↓ R (NG = -5.4 Ω, -1.5%; WG = -16.5 Ω, -4.5%) ↑ Xc (WG = + 2.2 Ω, + 6.3%)
Campa F, 2021	Italy	Male	36	67.4 ± 5.1	12 weeks, 3x	7 exercises 3 × 12	101 Anniversary, AKERN Systems, Florence, Italy; to hand technology at 50 kHz	↑ PhA (ST = + 0.3°,+4.6%; ET = + 0.3°,+4.6%) ↓ R (ST = -19.1 Ω, -3.2%) ↑ Xc (ST = + 0.9 Ω, 2.5%)

Note: N.A. = Not avaiable; CLG = Group with constant load; PLG = Group with pyramidal load; G1S = Group with one series per exercise; G3S = Group with three sets per exercise; NG = Group with narrow repetition zone training; WZ = Group with wide repetition zone training; ST = suspension training; ET = Elastic tube training; PhA = Phase Angle; R = Resistance; Xc = Reactance.

**Table 2:** Changes in bioelectrical impedance parameters from studies selected for the meta-analysis

First Author, Year	Group (n)	PhA at Pre (Mean ± SD)	PhA at Post	ES	R at Pre (Mean ± SD)	R after Post	ES	Xc at Pre (Mean ± SD)	Xc after Post	ES
Tomeleri CM, 2018	TG (24) CG (22)	5.4 ± 0.6 5.6 ± 0.5	5.8 ± 0.7*§ 5.4 ± 0.5*	+ 0.61 − 0.40	560.3 ± 56.1 579.8 ± 71.5	547.1 ± 56.7*§ 584.1 ± 70.3	− 0.23 + 0.06	53.3 ± 7.9 57.0 ± 9.8	55.9 ± 8.7* 55.4 ± 8.3	+ 0.18 -0.15
Cunha PM, 2018	G1S (20) G3S (20) CG (22)	5.8 ± 0.5 5.5 ± 0.5 5.6 ± 0.6	6.1 ± 0.4*§ 5.9 ± 0.6*§ 5.3 ± 0.5*	+ 0.45 + 0.73 − 0.56	552.6 ± 51.7 603.5 ± 59.8 589.0 ± 74.3	528.1 ± 45.6*§ 570.9 ± 58.5*§ 594.6 ± 71.1	− 0.50 − 0.55 + 0.08	56.3 ± 5.3 58.1 ± 7.5 58.6 ± 10.1	59.4 ± 4.5*§ 61.8 ± 8.4*§ 55.8 ± 8.6	+ 0.64 + 0.48 − 0.33
Campa F, 2018	ST (15) CG (15)	5.6 ± 0.4 5.6 ± 0.4	5.9 ± 0.5* 5.5 ± 0.5	+ 1.26 − 0.60	555.2 ± 46.9 536.2 ± 46.7	540.2 ± 49.2* 540.7 ± 46.2	− 0.94 + 0.06	53.6 ± 4.1 52.3 ± 7.9	57.1 ± 4.5* 51.2 ± 7.2	+ 1.41 − 0.20
Ribeiro AS, 2017	CLG (25) PLG (26) CG (26)	5.6 ± 0.4 5.4 ± 0.6 5.5 ± 0.5	5.7 ± 0.5*§ 5.6 ± 0.6*§ 5.4 ± 0.4	+ 0.30 + 0.35 − 0.17	586.5 ± 65.2 574.7 ± 59.5 580.5 ± 80.9	564.9 ± 74.2*§ 559.8 ± 60.4*§ 587.0 ± 79.2	− 0.31 − 0.25 + 0.08	55.6 ± 6.2 53.9 ± 7.7 55.7 ± 9.1	57.5 ± 7.6*§ 57.9 ± 8.4*§ 54.6 ± 8.0	+ 0.28 + 0.50 − 0.13
Souza MF, 2017	GT (19) CG (22)	5.5 ± 0.5 5.6 ± 0.5	5.8 ± 0.6* 5.4 ± 0.6	+ 0.91 − 0.60	591.2 ± 75.0 585.2 ± 74.2	572.1 ± 69.2* 590.3 ± 74.3	− 0.32 + 0.09	57.2 ± 8.2 57.6 ± 9.5	58.8 ± 9.5 56.5 ± 8.3	+ 0.31 − 0.40
Dos Santos L, 2020	NG (19) WG (18) CG (18)	5.4 ± 0.7 5.4 ± 0.9 5.6 ± 0.9	5.7 ± 0.6*§ 6.0 ± 0.7*§ 5.2 ± 0.6	+ 0.41 + 0.48 − 0.22	564.0 ± 44.8 574.5 ± 55.8 572.1 ± 72.3	555.4 ± 51.2*§ 549.2 ± 60.5*§ 587.2 ± 75.7*	− 0.19 − 0.45 + 0.21	53.6 ± 5.1 54.5 ± 6.8 54.7 ± 5.0	55.7 ± 5.9 57.8 ± 5.4* 53.6 ± 7.0	+ 0.41 + 0.48 − 0.22
Campa F, 2021	ST (11) ET (11) CG (11)	6.5 ± 0.6 6.5 ± 0.7 6.1 ± 0.6	6.8 ± 0.7*§ 6.8 ± 0.8*§ 5.8 ± 0.4*	+ 0.62 + 0.40 − 0.40	467.0 ± 37.3 453.7 ± 70.4 457.8 ± 12.7	452.4 ± 37.2*§ 441.0 ± 70.4 480.0 ± 12.7	− 0.43 − 0.30 + 0.10	52.3 ± 8.0 50.8 ± 7.1 48.7 ± 4.2	54.0 ± 8.3*§ 51.6 ± 7.9 48.5 ± 3.4	+ 0.18 + 0.16 − 0.01

Note: *= p < 0.05 vs. pre-training; § = p < 0.05 vs. group control; n = Sample size; CG = Control group; TG = training group; CLG = Group with constant load; PLG = Group with pyramidal load; G1S = Group with a series per exercise; G3S = Group with three sets per exercise; ST = suspension training group; ET = Elastic training group; PhA = Phase Angle, expressed in degrees; Xc = Reactance, expressed in ohms; R = Resistance, expressed in ohms; ES = Effect Size.

### Impact of resistance training on bioimpedance parameters

**Fig. 2:**
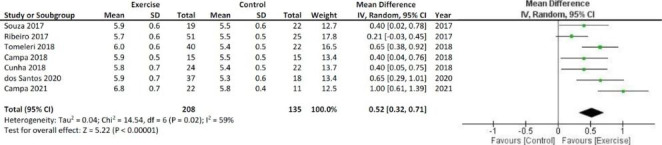
Meta-analysis and Forest Plot for the bioelectrical phase Angle expressed in degree. SD = standard deviation; CI = Confidence interval

**Fig. 3:**
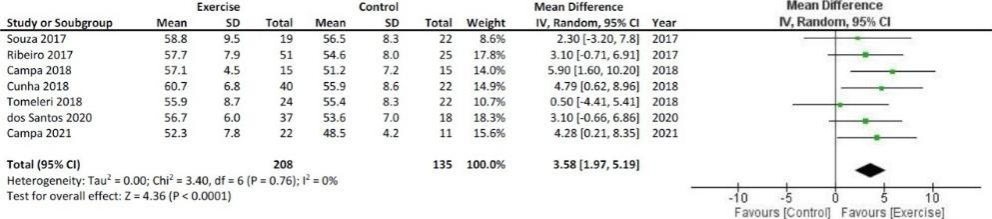
Meta-analysis and Forest Plot for the bioelectrical reactance expressed in ohms. SD = standard deviation; CI = Confidence interval

**Fig. 4:**
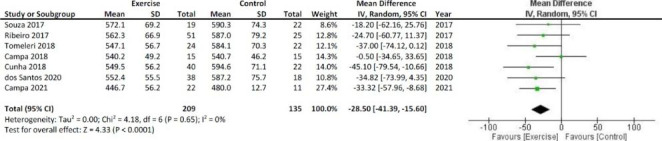
Meta-analysis and Forest Plot for the bioelectrical resistance expressed in ohms. SD = standard deviation; CI = Confidence interval

A fixed-effect model was used for all meta-analyzes. The results of the meta-analysis show significant increases in PhA and Xc, as shown in Figs. 2 and 3, respectively. The bioelectrical R decreased after the resistance training with respect to the inactivity period, as shown in Fig. 4.

**Fig. 5:**
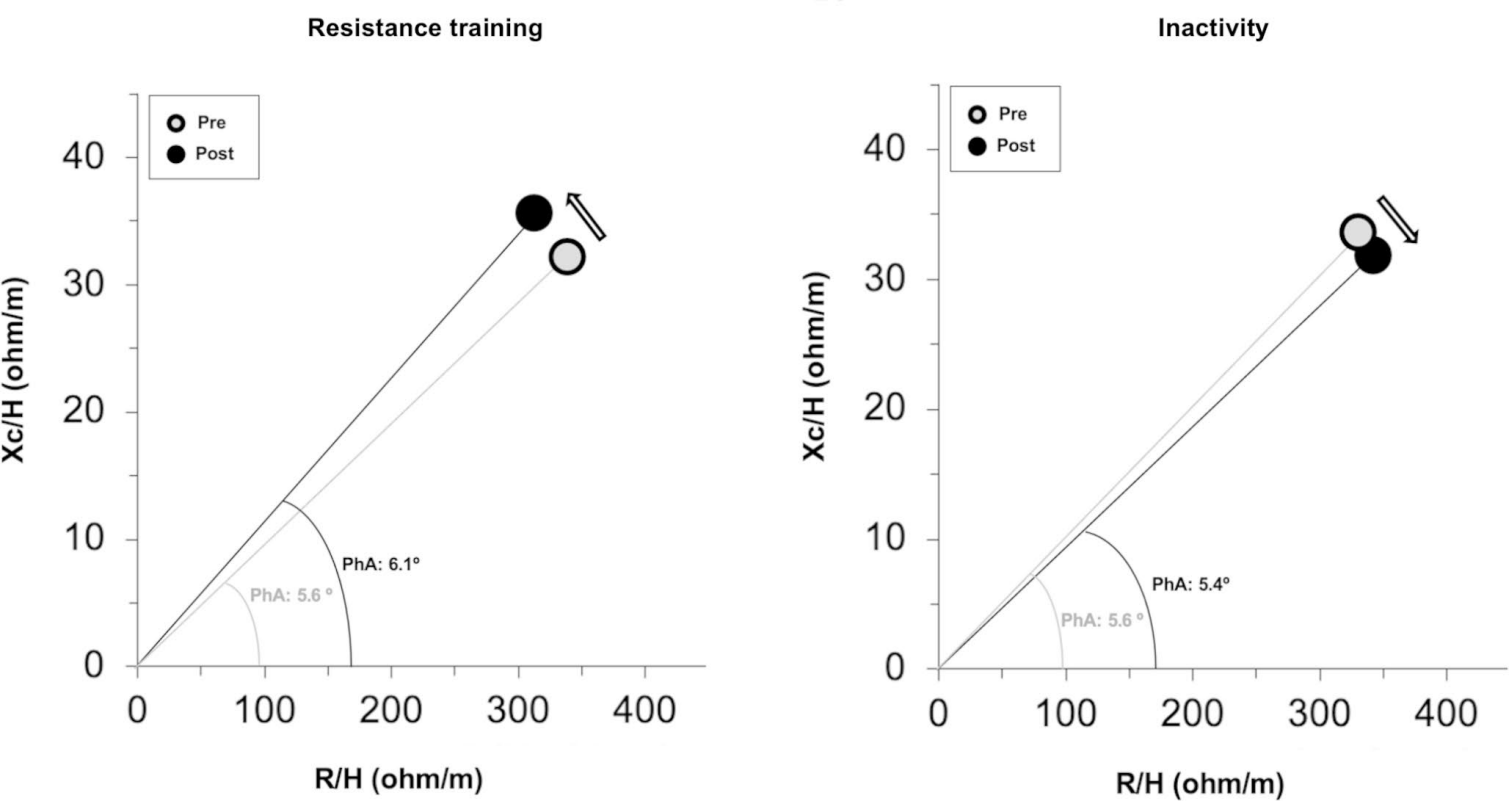
Description of the bioelectrical vector displacements in response to resistance training (left panel) and to inactivity (right panel) within the R-Xc graph. Data were calculated from the mean values of the selected studies

Figure 5 describes the mean bioelectrical vector displacement induced by resistance training or the inactivity period. The changes in BIVA patterns (vector length and direction) were calculated from the mean values (Table 2) of the selected studies and plotted into the R-Xc graph. After resistance training, a leftward vector displacement, representing an increase in PhA, occurred as a result of the Xc increase and R decrease (Fig. 5). The control group experienced a rightward vector displacement and therefore a PhA decrement, as a result of the Xc decrease and R increase (Fig. 5).

## Discussion

The present systematic review with meta-analysis aimed to determine how bioelectrical R, Xc, and PhA change after a resistance training program with respect to an inactivity period in older people. Resistance training has been shown to effectively increase Xc, decrease R, and the combination of these changes resulted in PhA increases. According to BIVA, resistance-trained people experienced a leftward vector displacement, while inactivity induced a rightward vector displacement within the R-Xc graph. To our knowledge, this was the first meta-analysis that investigated the effects of resistance training, compared to inactive control groups, on raw bioelectrical parameters.

**Fig. 6:**
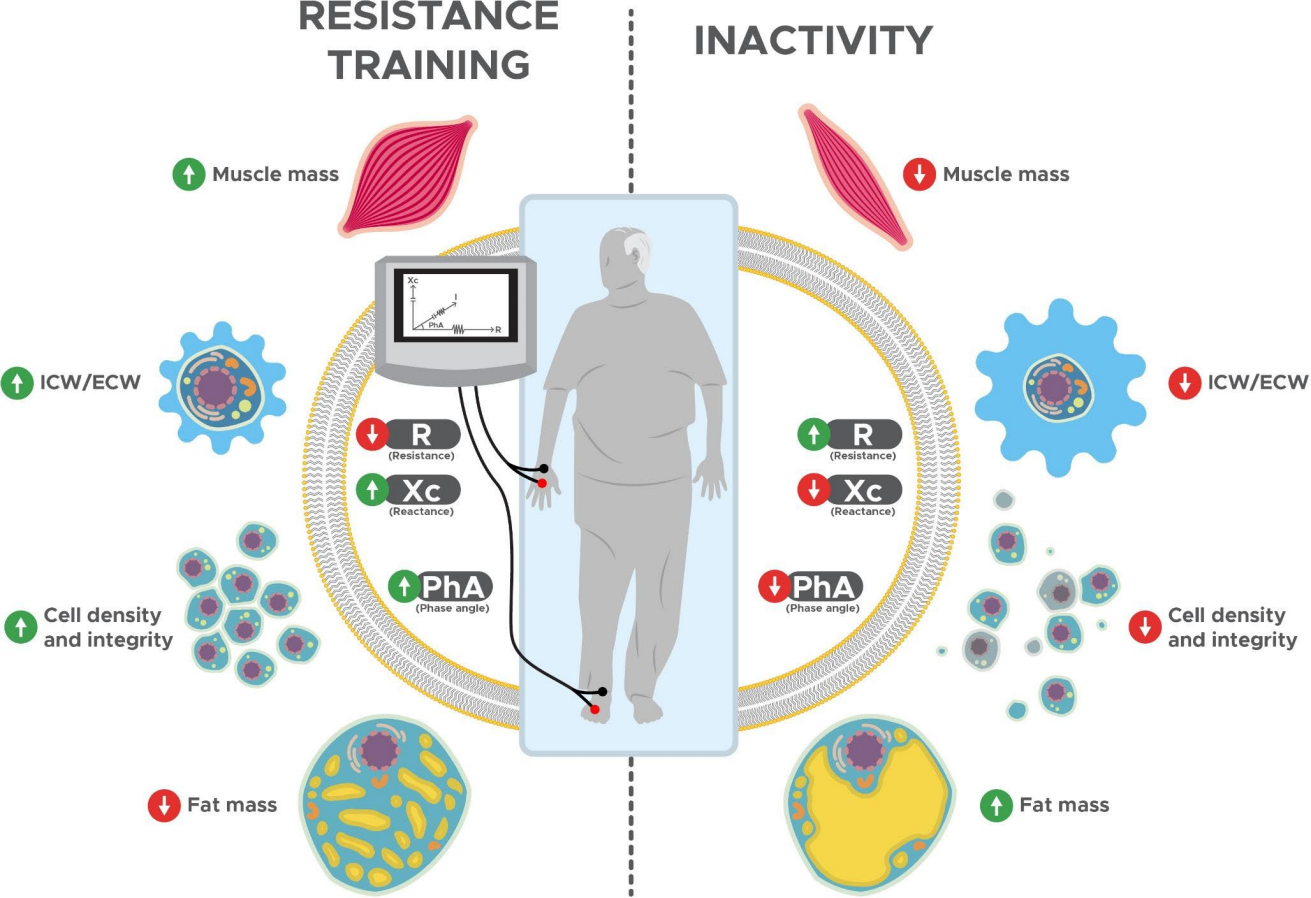
Schematization of the change in bioelectrical parameters and the possible remodeling in body composition related to resistance training or inactivity

In the elected studies, changes in bioimpedance parameters were associated with a body composition remodeling. After the resistance training program, five of the seven randomized controlled trials showed a reduction between 1.3 and 7% of total body fat [28, 33, 35, 37], while four studies reported increases in intracellular water content between 3.1 and 12.4% [34, 35, 37, 38] and three studies assessed muscle tissue increments between 1.3 and 6.6% [35, 37, 38]. However, no reference methods for assessing muscle mass or fluids were included in the aforementioned studies and some of these changes are within the technical errors of the reference methods [34, 35, 38]. Starting from the fluids content and its distribution among intra and extra cellular compartments, adaptations at the molecular (e.g., body fat and water) and cellular level (e.g., intracellular and extracellular water) result in tissue components (e.g., lean soft and muscle tissue) [14, 43]. BIA is based on the theory that the human body is a circuit formed by resistors, represented by body fluids and their electrolytes, while cell membranes and tissue interfaces act as capacitors [8, 9, 44]. Muscle tissue is basically composed of water and electrolytes and is a good electrical conductor, while cell membranes, body fat, and bones are composed of anhydrous materials with reduced conductivity [10, 44]. Considering that R depends directly on tissue hydration and is inversely associated with total body water [7], the reduction in favor of the resistance training group can be a result of both increases in intracellular hydration and then in muscle tissue. Resistance training practice causes increases in Xc, which can be justified by the increase in intracellular water, as mentioned previously. A possible explanation for these adaptations is the increase in muscle proteins induced by exercise [45, 46]. In addition to the molecular adaptations associated with resistance training, two of the selected studies reported improvements in inflammatory biomarkers and oxidative stress with improved muscle quality [34, 35], whereas one study showed and increased muscle strength [38]. Although the results are promising, further studies are needed to determine the effectiveness of resistance training for these variables. The combination of these responses to the resistance training resulted in an increase of the PhA, which can also be evaluated through the bioimpedance vector analysis. In this regard, the direction of the vector was informative of the PhA changes, for which leftward and rightward displacements occurred after the resistance training or inactivity, respectively [28, 33, 36, 37]. Previous studies involving dilution techniques as reference method showed that PhA can be considered a valid predictor of the intracellular-to-extracellular water ratio, and its increment is associated with higher cell density and integrity in healthy older subjects [12, 19, 26, 47, 48]. In contrast, the combined effect of aging with inactivity was associated with a decrement of PhA in the participants included in the control groups of the selected studies. Figure 6 schematizes the change in bioelectrical parameters and the possible remodeling in body composition related to resistance training or inactivity. However, although change in intracellular-to-extracellular water ratio, cell integrity and density markers, and muscle mass have been previously correlated with bioelectrical proprieties [26, 41, 49], a direct relationship between BIA-derived parameters and body fat has not been documented yet. Monitoring PhA may therefore identify training-related adaptations in response to exercise, for which the resistance training appears to be an effective practice.

All the studies included in this meta-analysis respected the basic principles of resistance training, applying a resistance exercise with a gradual progression in the load, following the required frequency training and recovery times [2]. However, although the practice of resistance training clearly induced improvements in PhA, the magnitude of its change was affected by the procedures by which the exercise was administered. In this regard, training with weight machines and suspension tools with a wide pyramid system, and performing more than one set for each exercise have induced major changes in the bioelectrical parameters [28, 33, 34, 36, 38]. On the contrary, resistance training exercises using bodyweight or elastic tubes as resistance induced adaptations of a lower magnitude, still being able to counteract the effects of aging added to inactivity [33]. Two mechanisms associated with resistance training, muscle damage and metabolic stress, could be potentially responsible for generating adaptations in the inflammatory response, resulting in localized ruptures in the structures of muscle cells, changes in the hormonal environment, and edema [50]. Knowing that resistance training can be performed indoors or outdoors and with a wide range of tools, the challenge is how to motivate older adults to follow exercise programs while also paying attention to their body composition characteristics.

Only randomized controlled trial studies were included in this study, which made it possible to meta-analyze the evidence with the highest degree of quality available in the literature. This was possible due to the elaboration of the research protocol, based on solid driving recommendations, enabling the determination of adequate eligibility criteria and consistent search strategies. In addition, the use of a validated instrument for the analysis of the overall quality of the evidence, made it possible to define clinical recommendations regarding the effectiveness of resistance training in inducing adaptations on the BIA-derived parameters. From a practical point of view, it is important to suggest what might be the minimum change in PhA needed to indicate a beneficial effect of resistance training in older people. In this regard, two aspects should be considered. First, the absence of PhA variation can also be considered a positive effect of resistance training, as inactivity causes reductions in PhA during aging [20]. Second, previous studies using the same BIA technologies reported a test-retest coefficient of variation ranging from 0.3 to 0.6% and from 0.9 to 1.5% for R and Xc, respectively [51, 52]. Therefore, to have confidence and conclude a beneficial effect of resistance training in elderly individuals, an increase of 0.5 degree in PhA should be considered as a minimum magnitude.

This systematic review with meta-analysis had some limitations. The low number of male participants included in elected studies precluded the exploration of gender as an influential factor. Another limitation was the inclusion of only healthy individuals, since there is no other available data in the current literature. Therefore, the results verified here should not be extrapolated to older adults with pathological conditions or diseases, as well as different populations. Lastly, direct comparisons of changes in absolute bioelectrical measurements should be avoided because all bioimpedance devices do not provide same values [53, 54].

Considering that any form of resistance training produced improvements in bioelectrical parameters, future studies are needed to determine which equipment and which pyramidal strategies can induce major changes in body composition in older adults. Finally, wide-scale of longitudinal studies are needed to better clarify the predictive role of R, Xc, and PhA in assessing changes of body composition parameters obtained from reference methods.

## Conclusions

In conclusion, the present results provide valid evidence that resistance training promotes increases of bioelectrical PhA and Xc, while inducing reductions of R during aging. Regarding BIVA, resistance-trained older adults can experience a leftward vector displacement and a PhA increase, while inactivity may result in a rightward vector displacement and a PhA reduction. This meta-analysis contributes to decision making regarding the use of BIA in monitoring body composition remodeling associated with the resistance training practice.

## Supplementary Table

**Table S1:** Methodological Assessment with Rosendal Scale

Citation	A clear description of the inclusion and exclusion criteria was provided	The trials were randomized	Treatment order was counterbalanced	The method used to generate the random allocation sequence was described	Sample size was justified	Attempts were made to control and/or monitor pre-trial conditions	Design incorporated measures of important baseline variables	Subjects were blinded	Investigators were blinded	Methods and successfulness of blinding were described	Details were provided regarding the inability of a subject to complete study requirements	Statistical methods described	Primary outcome measurement and variability reported	Results of statistical comparisons reported	Methods used to assess adverse effects described	Reproducibility of the primary outcome measure(s) was reported	A familiarization of the performance test was conducted	Total Score (%)
Ribeiro et al.	1	1	0	1	0	1	0	NA	NA	NA	1	1	1	1	0	1	0	64
Souza et al.	1	1	0	1	0	1	0	NA	NA	NA	1	1	1	1	0	1	0	64
Campa et al.	1	1	0	1	1	1	1	NA	NA	NA	NA	1	1	1	NA	1	0	83
Cunha et al.	1	1	0	1	0	1	1	NA	NA	NA	NA	1	1	1	NA	1	0	75
Tomeleri et al.	1	1	0	1	0	1	1	NA	NA	NA	1	1	1	1	0	1	0	71
Dos Santos et al.	1	1	0	0	1	1	1	NA	NA	NA	1	1	0	1	1	1	0	71
Campa et al.	1	1	0	1	1	1	1	NA	NA	NA	1	1	1	1	0	0	0	71

## Abbreviations

BIA: Bioelectrical impedance analysis
BIVA: Bioelectrical impedance vector analysis
PhA: Phase angle
R: Resistance
Xc: Reactance
